# Surgical options in extensive burns management

**Published:** 2012

**Authors:** C Niţescu, DR Calotă, IP Florescu, I Lascăr

**Affiliations:** * Clinical Emergency Hospital for Plastic, Reconstructive and Burns Surgery, Bucharest, Romania; ** Department of Plastic Surgery and Reconstructive Microsurgery, Bagdasar-Arseni Emergency Hospital, Bucharest, Romania; *** Department of Plastic Surgery and Reconstructive Microsurgery, Clinical Emergency Hospital, Bucharest, Romania; "Carol Davila" University of Medicine and Pharmacy Bucharest, Romania

**Keywords:** surgical burn treatment, early excision, skin graft, allograft

## Abstract

**Background – hypothesis and objective:** In the past decades, extensive burn care has improved to the extent that burn victims can now frequently survive. Current treatment of a severe burned patient extends beyond the preservation of life and function; the ultimate goal is the return of survivors, as full participants, back to their families, work field and communities.

**Methods:** This paper is based on our experience in treating patients with extensive burns. We had two panels as follows: 148 subjects with extensive burns in one original retrospective study (2002-2009) and other 47 new patients with extensive burns enrolled in other original prospective study (2010-2011). We selected the subjects with extensive burns who received allotransplant (n = 59). Our study aimed to identify and quantify the main psychosocial difficulties in patients with extensive burns who received allotransplant, also taking into account the surgical procedures applied in each case.

**Results:** One of the major problems a surgeon faces is the nature of the decision he has to make regarding a treatment (conservative versus operative), based on the exact determination of burn wound depth. In case of early excision, deep lesions and suitable grafting represent the chosen surgical treatment in our center. The benefits of skin allografts are well known due to their contribution to burn management.

**Conclusions & discussion:** This paper is designed as a guideline and instruction manual to help those with less experience through particular situations in surgical burn care. Early excision and immediate coverage of the burn wounds represent nowadays the standard care for extensive burned patients. For patients with massive thermal injury, temporary coverage with allografts is essential. The use of allograft has multiple benefits because it plays both as physiologic and mechanical barriers.

## Introduction

In the past decades, extensive burn care has improved to the extent that burn victims have frequently a chance of survival. Current treatment of a severe burned patient extends beyond the preservation of life and function and, in our opinion, the ultimate goal is to return the survivors, back to their families, work field and communities. Extensive burns represent not only a very serious illness with potentially fatal complications, but also traumatic events, with significant potential for development of complex psychological issues, with multiple ramifications.

With the remarkable progress in the field of burns treatment, the outcome of extensive burns (Total Burned Surface Area, TBSA >25%) improved significantly, with recorded cases of survival even after 95% TBSA burns [**1,2**]. Patients with severe burns should be treated in a specialized burn facility after initially being assessed and treated in an emergency department. Generally, patients with burns over 45% TBSA benefit of allotransplant (using free split-thickness skin grafts [STSG] stored in skin bank or taken from donors) for serial excision-grafting interventions. This treatment is expensive, which implicitly, involves considerable human and material resources. Moreover, patients are discharged after 60-80 days of hospitalization (it is estimated one day of hospitalization/percentage of TBSA).

The objective of this paper was the consideration of the complex surgical treatment of extensive burned patients. It can also be used as a guideline regarding the surgical treatment of acute burns. This paper is mainly based on our personal experience in treating patients with severe burn injuries.

## Methods

One retrospective study, conducted between 2002-2009 in the ICU units from Bucharest Emergency Hospital for Plastic Reconstructive and Burns Surgery, and from Plastic Surgery and Reconstructive Microsurgery Department, Bagdasar-Arseni Emergency Hospital, was conducted in order to investigate the extensive burn treatments in these units. The study group was composed of 148 selected patients with burns of grade II B or III, with burn areas ≥25% TBSA. From this point, we started a prospective study intended to identify and quantify the main psychosocial difficulties in patients with extensive burns who received allotransplant, considering also the surgical procedures applied in each case. Thus, we selected patients with extensive burns (> 25% TBSA) who received allotransplant. Out of the 148 subject-panel with extensive burns in the retrospective study (2002-2009) and the 47 new enrolled patients with extensive burns in the prospective study (2010-2011), we selected all the subjects with extensive burns who received allotransplant (n = 59) but we eliminated the death cases (n = 18), in the end remaining 41 subjects. The age of our patients ranged from 21 to 57 years; of the 26 subjects, 15 were men (sex ratio M/F = 1.36); the causes of injuries being made by scalds and hot substances, fire and flame sources, as well as electrical sources. A relatively low but significant rate of admissions was performed due to self-induced burns. In 72% of all cases, immediate surgical interventions (escharotmies) were required. The mean numbers of surgical interventions were 1.8 +/- 1.4 per patient.

## Results

### Extensive burns management

One of the major problems a surgeon faces is the nature of the decision he has to make regarding a treatment (conservative versus operative treatment). In the case of an operative procedure, a decision is needed in order to determine when and how to excise the burn tissues and also to determine as accurate as possible the depth of the thermal lesions and thereby the extent of tissue involvement [**3-5**]. The vascular patency is a very sensitive indicator for the extent of the damaged tissues [**6**]. The currently accepted classification of acute burns describes three levels of injuries, based on a combination of the clinical estimation of depth and the outcome as follows: superficial partial thickness burns (Grade I, Grade IIa), deep partial thickness burns (deep dermal, Grade IIb) and full thickness burns (Grade 3) [**7,8**].

In our units, the serial clinical examination is still the golden standard to appreciate the depth of a burn wound areas - in seriously burned patients- that appeared, at first as partial thick burns that may come to be regarded within the next days as fully thickened.

### Escharotomy and limb decompression

The circular eschar of full thickness burn injury exerts a tourniquet effect on extremities, constricting the chest and the abdomen [**9,10**]. Classically, but not exclusively, this occurs mainly in circumferential full thickness burn injury. In extremities, failure to release the eschar leads to soft tissue hypoperfusion, deficient oxygenation and eventually necrosis. In the extremities, prompt recognition of this serious condition and immediate restoration of circulation is required. Experimental data suggests that muscles undergo irreversible damages in 4–12 h and nerves within 12–24 h [**11**]. Frequently, complications following this acute compartment syndrome occur due to inadequate escharotomies such as distal amputation and sepsis. In the last decade, the term escharotomy tends to be less preferred in favor of the concept “limb decompression” [**12**], so that it encourages the surgeon to have a high level of suspicion for impaired limb vascularity despite escharotomy and a low threshold for performing formal fasciotomies.

Constricting eschar around the thorax can lead to impaired ventilation and gas exchange abnormalities. If the eschars interest the abdominal wall compliance, an abdominal compartment syndrome may occur along with impaired cardiorespiratory function and reduced renal perfusion [**11**].

The escharotomy consists in division of the eschar, utilizing scalpel incisions down the long axis of the limb in mid-medial or mid-lateral lines through the dermis [**13**] or down to deep fascia [**12**], extending into unburned tissues and across joints [**14**]. Literature regarding the exact extent of these escharotomies is occasionally contradictory. Despite the insensate nature of the eschar, these procedures should be performed in an operating room under sterile procedure with an aseptic technique and the ability for hemostasis. Due to generalized edema and increased microvascular permeability [**15**], the compartment syndrome might occur in a burned limb as well as in the unburned limb of a patient with extensive burns [**16,17**]. In the cases of patients in which an abdominal compartment syndrome was suspected, decompression would include escharotomy and, in severe cases, laparotomy should be taken into consideration.

### Early excision and grafting

Before the era of early excision and grafting, burns were treated with removal of loose dead tissue (by serial debridements) and be applying sterile dressings. If infection was avoided, superficial burns would have healed within 2 weeks but deeper burns would have taken longer to heal. Full-thickness burns lost their eschars due to enzymatic debridement, enhanced by colonized bacteria. In such cases, split skin grafts were applied 3–6 weeks after the injury occurred over the underlying layer of granulation tissue. The rates of graft loss were high, so repeated attempts were often necessary in order to close the wound. In the past, when this treatment was applied, burn scars contractures and hypertrophic scars were inevitable results. The popularization of early burn wound excision was made in 1960 by Jackson [**18**] and in 1970 by Janzekovic [**19**].

Early excision removed all devitalized tissues, decreasing mortality, morbidity, bacterial colonization, length of hospital stay, time away from work and decrease costs [**20-23**]. Advances in anesthesia, intensive care, fluid resuscitation and use of blood derivates have made this type of surgical procedure safer. Advocates of early burn wound excision seek to modify the host inflammatory response to ameliorate SIRS and prevent organ dysfunction. Improved survival was noted in children with massive burns when treated in this manner [**24**] but must be applied cautiously in elderly populations with TBSA burns greater than 20% [**25**].

In the case of an early excision procedure, a decision is needed on when to excise the burn lesions and, of course, to determine accurately the depth of the wound and implicitly the extent of tissue involvement. There are still many controversies regarding when and how to excise but today it is widely accepted that if skin does not regenerate in a few weeks, morbidity and scarring will be severe. In fact, the trend in the treatment of deep dermal partial and full thickness burns leans toward very early excision and grafting in order to reduce the risk of infection, decrease scar formation, reduce hospital stay and decrease costs.

The excision timing is an area of controversy. Excision of as much of the burn wound as possible should be carried out whenever a patient is hemodynamically stable and the risks associated with the intervention would not lead to mortality. In cases with associated lesions such as inhalation injury, elderly patients or with cardiac or respiratory problems, special measures are required in order to decide when and how much to excise. Generally, we start to excise all deep dermal and full thickness burned areas within the first 72 hours of injury (between 48 and 72 hours). It has been proved that early excision is better than late excision, before high bacterial contamination occurs within 5-7 days after injury. After that, the incidence of sepsis and graft failure increases.

**Techniques.** Sequential layered tangential excision to viable bleeding points, even to fat, is the generally accepted technique. Excision of burn wounds to the fascia is reserved for extensive burns, where the risks of massive blood loss and the possibility of skin lost due to less vascularized grafts on fat may lead to higher mortality and morbidity.

Burn wounds may be treated surgically by: tangential excision, fascial excision or amputation.

• ***Tangential excision***. The principle is to remove all the necrotic tissue and to preserve viable dermis in the wound bed. This technique was based on the observation that deep donor sites for skin grafts could be successfully overgrafted with split thickness skin grafts [**26**]. One of the important advantages of this procedure is that the contours are better preserved, healing taking over faster and the length of hospital stay is reduced [**19**].
• ***Fascial excision ***is an alternative that is quicker and easier to perform and, above all, the degree of blood loss is low. However, it leads to contour defects and lymphedema. Moreover, fascial excision is indicated in full thickness burns, in which the underlying subcutaneous tissues are damaged. In addition, in life-threatening invasive wounds, in the presence of sepsis, especially when fungal organisms are involved, fascial excision should be considered.
• ***Amputation ***is applicable in unsalvageable limbs, in very deep burns and electrocutions. It eliminates the function as well as the burn; so it becomes an invalid procedure but should not be disregarded and still should be taken into account in electrical injuries and war wounds.

No matter the nature of the technique used, the burn wound should be excised until all the non-viable substances are removed and healthy bleeding tissue is evident, creating a viable wound bed. Tangential excision can result in copious blood loss. Blood product use can be minimized by reducing intraoperative bleeding and reducing the transfusion trigger [**27**]. To minimize blood loss, various strategies have been employed but no comparative evidence exists between the different approaches. Consequently, having a strategy would significantly reduce transfusion requirements [**28**]. In appropriate cases, limb wounds can be excised under tourniquet [**27,29**]. This requires experience in order not to excise healthy tissue. Adrenalin infiltration of the wound bed prior to excision and adrenalin soaks placed upon the excised wound produce vasoconstriction and reduced blood loss with no systemic effects [**30,31**]. Systemic recombinant activated factor VII (rVIIa) reduces blood loss and transfusion requirements in the cases of excision and grafting of extensive burns, with no increased rate of thromboembolic complications [**32**].

Regarding the time of intervention, it has been shown that excision of the burn within the first 24 h after the accident would significantly reduce blood loss, with no increase in mortality in a children population [**33**], whereas in adult populations, meta-analysis of available data confirms that early excision reduces mortality and length of hospital stay in patients without inhalational injury [**34**]. There are no clear data regarding when and in what manner excisions should be performed. Complete early burn wounds excision in 24 h with wound coverage is a substantial logistical undertaking in the extensive burned patient and should be performed by a team of surgeons in a burn center.

In deep facial burns, we prefer a conservative approach by use of silver sulfadiazine and some consideration should be given to performing tarsorrhaphies for ocular protection. Full thickness burns will be excised and grafted with unmeshed skin grafts according to aesthetic units in the face.

Hand burns should be dressed with silver sulfadiazine and elevated with the hand splinted in the position of safe immobilization; consideration can be given to K-wire fixation of the digits to maintain joint posture [**35**]. Deep partial thickness burns covering the hand area will be debrided tangentially and covered with unmeshed skin grafts. Full thickness burns will be excised and grafted with unmeshed skin.

Gold standard **wound coverage** is an autograft applied in sheets on sensitive areas such as hands and face or meshed in order to cover larger areas. Meshed autografts increase the surface area that should be covered. We generally use expanded meshed autografts to cover wounds in cases of sufficient donor sites. Expansion rate of grafts ranges from 1:1.5 to 1:6 [**36**]. Expansion rates higher than 1:3 heal in a suboptimal manner leading to contractures and thin, easily injured skin. Therefore, we like to combine these large mashed skin grafts in combination with allografts (the so-called “sandwich technique” in order to improve the aesthetic and functional outcome).

In smaller burns, less than 40% TBSA, donor sites for autografts are seldom a problem unless the patient is at risk of surgical complication because of age, comorbidities or coagulopathy. In massive burns, unusual donor sites could be used, such as the axillae, scrotum, mons pubis and even the soles of the feet [**26**].

Patients with greater burns have a lack of available donor sites. Thus, we use skin allografts as a temporary cover. The allograft decreases the size of open wounds until autograft becomes available, as the partial thickness burn area is healed or previously harvested from donor sites. This temporary covering helps us control the wound infection, prevents wound contracture, and is less painful. An essential component in the use of allograft is the ability to store and preserve the skin for its later use under standardized conditions.

### Allografts

Viable human allograft skin prelevated from cadaveric sources has proved to be a very effective material to cover tangentially excised deep second- or third-degree burn wounds when the amount of autografts has been insufficient. Allograft cryopreserved skin plays both a mechanical and a physiological barrier, its use has been proved to decrease the loss of fluids, protein and heat through the burn wound, as well as preventing wound colonization with bacteria and, eventually, infection.

In patients with burns over 35% TBSA donor sites are soon exhausted. In such cases, allografts are used as temporary measures to seal the wounds whilst donor sites recover prior to regrafting. There are several methods which combine autografts with allografts: the Alexander technique [**37**] uses autograft meshed 1:6 “sandwiched” with allograft meshed 1:1.5 or 1:2 to help seal the wound post-operatively. The Meek technique [**38**] expands available autograft by using it in a postage stamp format.

In all cases when autografts are not available, allografts play an important role. Allografts can be fresh, cryopreserved or stored in glycerol (lyophilization). Allograft effectively seals the wound in the immediate post-excision phase, reducing heat loss, exudates and ameliorates the hypermetabolic response. Disadvantages associated with allograft include the inevitable rejection and the risk of infection present with all transplanted tissue.

In extensive burned patients, serial excision is a universally accepted approach. In serial excision, in each operative session as much as 20% TBSA of the burn wound is excised, with the aim to excise and cover the burn in a timely fashion. Clinical full thickness burns are excised first in order to avoid excising potentially viable tissue. In massive burns, large areas such as the posterior trunk, anterior trunk and lower limbs are excised and grafted first. If all these are involved, then an appropriate order may be back first, trunk second and limbs third.

Skin allografts have been used in medical practice for more than a century and are available in several forms. A fresh skin allograft is believed to be the best, [**39**] but it has limited resources and can be difficult to obtain. Cryopreserved and glycerol-preserved allografts (GPA) are other forms of skin allografts that are harvested, processed, and can be stored for a long time. This makes them more readily available compared with fresh skin allografts.

For many decades, the benefits of skin allografts as biological dressings are well known for their contribution in burn management. These benefits include protection of the wound bed against desiccation, colonization with bacteria, heat, electrolytes, and protein loss by creating a physiological as well as mechanical barrier. Fresh cadaver allografts are still considered to be the gold standard skin substitute by several authors [**39,40**] but their scarce availability has severely impeded their use. The advantages of GPA compared with cryopreserved and fresh allograft preparations have enhanced revascularization of the wound bed by promoting neo-vascularisation, low antigenicity resulting in a decreased rejection reaction, [**40,41**] marked shortening of healing time, [**40-42**] enhanced wound epithelialisation, prevention of wound bed deterioration and secondary necrosis, [**41,44**] and reduction in the risk of transmissible disease [**39**].

### Skin substitutes and future strategies

Skin substitutes provide temporary physiological wound closure, preventing desiccation, fluid loss and reducing pain prior to spontaneous wound healing or autografting. In addition, they can be used in association with allograft in the treatment of large partial thickness burns and for the dressing of large donor sites. Nevertheless, due to their synthetic nature they remain a possible trigger for infection [**45**] and are expensive.

Biobrane® is a biosynthetic dressing composed of a nylon mesh that is bonded to a thin silicone membrane coated with porcine polypeptides. It is used as a temporary covering for clean, debrided superficial and mid-dermal burns [**46**] and has also been utilized as an adherent dressing over meshed autograft. It is designed to adhere to the wound base and it has been proved that Biobrane® is superior to 1% silver sulfadiazine in treating pediatric partial thickness injury [**47,48**].

Integra® is a dermal regeneration template composed of two layers. The outer layer is a removable silicon sheet, the inner layer is a bovine collagen matrix designed to act as a dermal analogue. This dermal analogue is to be vascularized, forming a neo-dermis within 4 weeks [**49**]. Once formed the outer silicon layer is removed and a thin autograft placed onto the neo-dermis. Its use in acute burn resurfacing with cultured keratinocytes has also been reported [**50**]. Matriderm® is similar in concept to Integra® but is marketed with the ability to place a graft at the first procedure.

AlloDerm® is an acellular dermal regeneration matrix derived from human skin developed on the observation that acellular dermal matrices do not undergo rejection. It is incorporated into the patient and a thin split thickness skin graft is placed upon it at the time of the index procedure. AlloDerm® is similar in concept to Integra® but is more appropriate in patients where a one-stage procedure is beneficial [**51**] and can be used acutely [**52**].

Since 1981, it was possible to culture large numbers of keratinocytes from a small sample of autologous skin prelevated for burn wound resurfacing [**53**]. Transfer of confluent sheets of cultured epithelial autograft (CEA) is expensive, exquisitely sensitive to infection with variable graft take rates, but it can be life saving in 60–70% TBSA burns [**54**]. Methods to import stem cells into wounds are in development [**55**]. There are some controversies regarding how to use CEA. Cultured keratinocytes have been sprayed onto wounds. This technique is used in conjunction with meshed autograft and has the purpose to decrease healing time while maximizing CEA impregnated sheets [**56**], which have also been recently utilized in similar circumstances.

Enzymatic debridement of the burn wound has obvious logistical advantages. It can be performed in the immediate post-injury period by emergency clinicians, can preserve more dermic tissue than surgical excision, reduce the requirement for limb decompression, and decrease blood loss. Various agents have been or are in the process of being examined and despite some encouraging results in children with non-infected partial thickness injuries [**57**] none are as widespread used. [**58**].

In selected cases, the use of allogenic keratinocytes is indicated to cover skin donor sites, because these will decrease the size of open wounds and, consequently, will decrease the healing time of previously harvested donor sites. The impact on the course of disease and its outcome is not yet decided. Similarly, the impact of the use of anabolic and anticatabolic agents is still unknown.

## Discussion

The early excision and immediate coverage of the burn wounds represent now the standard of care for extensive burned patients. In patients with massive thermal injury, temporary coverage with skin donated by another person (allograft) is essential. The use of allograft has the below-listed benefits:

• reduction of water, electrolyte and protein loss;
• decrease in energy requirements;
• minimizing the wound infection rates;
• decrease pain;
• conservation of autografts;
• improved general condition of the patient.

Human allografts are used as a split-thickness graft after being procured from organ donors [**59-61**]. When used fresh or in cryopreserved state, allografts vascularize and remain the “gold standard” for temporary wound closures. They can be refrigerated for up to 7 days, but must be stored in frozen places for extended periods. In addition, allografts can be used in a non-viable state after preservation in glycerol or in lyophilization. However, most literature data describes best results when it is used in a viable state. The epidermal component provides a barrier until rejected by the host in 3-4 weeks. Homograft, another term for human allograft, can only be obtained from a tissue bank, as strict protocols are required for harvesting and storage. Donors must be rigidly screened for potential viral and bacterial disease to avoid any risk of transmitting them. Therefore, the product is in limited supply and very expensive. The primary indication for use is to cover a large excised burn wound until an autogenous skin or a permanent skin substitute becomes available.

The main principles in the management of burn injuries in a specialized burn unit are the control of pain and infection, to provision and maintenance of a moist environment, and the prevention of heat, fluid and protein loss. Autograft is the ideal wound coverage, which can embody all these principles, but in severe burns there are not always available sufficient donor sites. Allografts, which are considered the best biological dressing, can be applied either temporarily until autografts become available, in conjunction with widely meshed autografts, or when complete healing and epithelialization of the burn wound occur [**62**]. The use of cryopreserved allografts in the treatment of burns is a well-known technique and offers several advantages such as the reduction of pain significantly, the prevention of wound desiccation, the prevention of water and electrolyte loss, and the decrease of protein leakage. Furthermore, allografts act as barriers to heat loss and exogenous bacteria. From a practical point of view, the burn wound is covered and the cells involved in the healing process are protected so that the body homeostasis is maintained [**63**].

Surgeons have an important role in the management of extensive burns, but only as part of a multidisciplinary team. The era of early excision, improved resuscitation and strategies to counter sepsis have improved survival and reduced morbidity. Nevertheless, future innovations are required in the field when it comes to the treatment of inhalational injury, to better skin quality coverage and minimizing the burden of leaving scars.

## Acknowledgements:

This work is partially supported by the Operational Programme Human Resources Development (HRD) funded by European Social Fund and the Romanian Government Decision no. SOP 64 153.

**Disclosure:** None.
